# mRNA expression of the DNA replication-initiation proteins in epithelial dysplasia and squamous cell carcinoma of the tongue

**DOI:** 10.1186/1471-2407-8-395

**Published:** 2008-12-30

**Authors:** Jian-na Li, Chong-jin Feng, Yong-jun Lu, Hui-jun Li, Zheng Tu, Gui-qing Liao, Chun Liang

**Affiliations:** 1 School of Life Sciences, Sun Yat-sen University, Guangzhou, PR China; 2 The First Affiliated Hospital, Sun Yat-sen University, Guangzhou, PR China; 3 Department of Biochemistry and Center for Cancer Research, Hong Kong University of Science and Technology, Clear Water Bay, Kowloon, Hong Kong, PR China; 4 Department of Oral and Maxillofacial Surgery, Guanghua College of Stomatology, Sun Yat-sen University, Guangzhou, PR China

## Abstract

**Background:**

The tongue squamous cell carcinomas (SCCs) are characterized by high mitotic activity, and early detection is desirable. Overexpression of the DNA replication-initiation proteins has been associated with dysplasia and malignancy. Our aim was to determine whether these proteins are useful biomarkers for assessing the development of tongue SCC.

**Methods:**

We analyzed the mRNA expression of CDC6, CDT1, MCM2 and CDC45 in formalin-fixed, paraffin-embedded benign and malignant tongue tissues using quantitative real-time PCR followed by statistical analysis.

**Results:**

We found that the expression levels are significantly higher in malignant SCC than mild precancerous epithelial dysplasia, and the expression levels in general increase with increasing grade of precancerous lesions from mild, moderate to severe epithelial dysplasia. CDC6 and CDC45 expression is dependent of the dysplasia grade and lymph node status. CDT1 expression is higher in severe dysplasia than in mild and moderate dysplasia. MCM2 expression is dependent of the dysplasia grade, lymph node status and clinical stage. The expression of the four genes is independent of tumor size or histological grade. A simple linear regression analysis revealed a linear increase in the mRNA levels of the four genes from the mild to severe dysplasia and SCC. A strong association was established between CDC6 and CDT1, and between MCM2 and CDC45 expression. The nonparametric receiver operating characteristic analysis suggested that MCM2 and CDC45 had a higher accuracy than CDC6 and CDT1 for distinguishing dysplasia from tongue SCC.

**Conclusion:**

These proteins can be used as biomarkers to distinguish precancerous dysplasia from SCC and are useful for early detection and diagnosis of SCC as an adjunct to clinicopathological parameters.

## Background

In eukaryotic cells, precise DNA replication is accomplished by licensing and activation of replication origins so that the genome is duplicated exactly once per cell cycle [1]. The *cis*-acting replicators are recognized by origin recognition complex (ORC), which recruits the loading factors, NOC3 [2], CDC6 and CDT1, thereby promoting the loading of the hexameric minichromosome maintenance (MCM) protein complex onto replicators, forming the pre-replication complex (pre-RC). In late G_1 _phase, CDC6 and CDT1 are dissociated from replicators and replaced by CDC45, forming the pre-initiation complex (pre-IC). As cells progress through S phase, MCM and CDC45 are displaced from the chromatin, leaving ORC and NOC3 on the replicators to form the post-replication complex (post-RC) [1,2]. Deregulation of the expression of DNA replication-initiation proteins results in genomic instability and contributes to the malignant transformation of cells [3].

In an adult human body, the vast majority of the cells has differentiated and ceased cell division, and only a small population of cells is proliferating in some self-renewing tissues. Some DNA replication-initiation proteins are expressed only in malignant and precancerous cells but not in nonproliferating, differentiated and replicative senescent human cells [4]. Therefore, the members of the DNA replication-initiation proteins are excellent biomarkers for cell proliferation [5-8] and targets for anticancer drugs [9].

More than 50% of head and neck cancers arise from the oral cavity, of which over 90% are squamous cell carcinomas (SCCs), with the majority representing SCC of the tongue [10]. Many oral carcinomas had pre-cancerous lesions including dysplasia. Although the oral cavity is easily accessible for clinical examination and biopsies, precancerous lesions are often missed, and oral cancers are usually diagnosed at late stages. Therefore, many patients still face poor prognosis. Currently only the gold standard of histological examination of a scalpel biopsy or surgical specimen can provide a definitive diagnosis. Early detection would offer better treatment, increased survival, and improved quality of life with reduced need for aggressive treatments or disfiguring surgery.

Studies in the last decade have demonstrated some of the most frequent molecular changes found in oral cancers, such as P53, P16, Cyclin D1, Ki67, and PCNA by using immunohistochemistry [11,12]. Since aberrant overexpression of the replication initiation machinery is found in many types of cancers, CDC6, CDT1, MCM2 and CDC45 may also be useful markers for assessing the type and degree of oral pre-malignant as well as malignant lesions. The purpose of the present study was to test the hypothesis that DNA replication-initiation proteins are useful biomarkers for early detection and diagnosis of SCC of the tongue and to elucidate the relationship between the expression of replication-initiation proteins and clinical characteristics using quantitative real-time PCR (QRT-PCR) assays. In addition, we assessed the correlation between the expression level of CDC6 and CDT1, and between CDC45 and MCM2 in SCC of the tongue.

## Methods

### Patient tissue specimens

Sixty-four patients with a median age of 54 years (range, 17 to 82 years) with SCC of the tongue who had surgery from August 2004 to July 2007 at the First Affiliated Hospital, Sun Yat-sen University, Guangzhou, China were included in this analysis. This study was approved by the Medical Ethical Committee of the First Affiliated Hospital, Sun Yat-sen University. All tumors were classified according to the standard of the American Joint Committee on Cancer (AJCC). Thirty-one epithelial dysplasia samples were used as the precancerous specimens, including 8 of mild epithelial dysplasia, 8 of moderate epithelial dysplasia and 15 of severe epithelial dysplasia. Histological grade, tumor stage and tumor size were examined and classified according to the International Union Against Cancer (UICC) scheme. All of the sites of distant metastases (M) were lymph nodes. The stage of primary carcinoma and patient characteristics are listed in Table 1. The surrounding normal tissues including stromal cells were largely removed from the surgical samples based on the gross morphology before formalin-fixation and paraffin-embedding. Serial section slices were then obtained, and some of the slices were used for pathological diagnosis microscopically by two pathologists after hematoxylin and eosin staining, while some other slices were used for QRT-PCR.

**Table 1:** Clinicopathological parameters and mRNA expression in the patients (n = 64)

**Characteristic**	**No.(%)**	**CDC6**	**CDT1**	**MCM2**	**CDC45**
Age (years)					
Median 54 (range 17–82)					
Sex					
Male	33(52%)	2.17E-03 ± 8.24E-04	1.10 ± 0.44	1.20 ± 0.24	2.17 ± 0.41
Female	31(48%)	6.28E-03 ± 1.97E-03	1.04 ± 0.34	1.28 ± 0.29	2.67 ± 0.89
Epithelial dysplasia (precancerous, n = 31)					
Mild	8(26%)	4.68E-04 ± 1.42E-04	0.64 ± 0.14	0.19 ± 0.05	0.35 ± 0.72
Moderate	8(26%)	6.59E-03 ± 3.20E-03	0.99 ± 0.20	1.03 ± 0.18	2.29 ± 0.85
Severe	15(48%)	8.28E-03 ± 3.40E-03	1.94 ± 0.36	2.48 ± 0.35	3.32 ± 0.54
Histological grade (SCC, n = 33)					
G1	13(40%)	6.87E-03 ± 2.67E-03	0.92 ± 0.19	1.15 ± 0.22	2.48 ± 0.82
G2	10(30%)	7.87E-03 ± 6.51E-04	0.72 ± 0.17	0.99 ± 0.30	1.97 ± 0.59
G3	10(30%)	8.61E-03 ± 1.30E-03	1.40 ± 0.30	1.73 ± 0.73	2.45 ± 0.13
T-stage					
T1	10(34%)	7.61E-03 ± 4.45E-03	1.01 ± 0.27	0.71 ± 0.20	3.17 ± 0.93
T2	19(66%)	5.02E-03 ± 7.62E-04	0.94 ± 0.14	1.56 ± 0.42	1.57 ± 0.44
Lymph node metastases					
N0	12(41%)	6.11E-04 ± 1.66E-04	0.74 ± 0.16	0.60 ± 0.15	1.27 ± 0.35
N1	11(38%)	3.50E-03 ± 1.02E-03	0.90 ± 0.15	1.03 ± 0.64	2.00 ± 0.52
N2	6(21%)	1.02E-02 ± 7.03E-03	1.28 ± 0.28	2.00 ± 0.42	3.54 ± 0.15
Clinical stage					
I	4(13%)	4.21E-04 ± 3.08E-04	0.89 ± 0.14	0.15 ± 0.04	1.13 ± 0.36
II	8(28%)	8.02E-04 ± 1.13E-04	0.74 ± 0.16	0.85 ± 0.21	1.55 ± 0.46
III	11(38%)	3.50E-03 ± 1.02E-03	1.07 ± 0.28	1.03 ± 0.64	2.01 ± 0.52
IV	6(21%)	1.02E-02 ± 7.03E-03	1.78 ± 0.82	2.00 ± 0.42	3.54 ± 0.15

T-stage (tumor size): Tl, < 2 cm; T2, 2 to 4 cm; T3 > 4 cm

G1, highly differentiated; G2, moderately differentiated; G3, poorly differentiated

Stage I, T1N0M0; stage II, T2N0M0; stage III, T3N0M0, T1-3N1-2M0; stage IV, T4N0-3M0

Shown is quantitative gene expression analysis by QRT-PCR in different formalin-fixed, paraffin-embedded specimens. Levels of CDC6, CDT1, MCM2 and CDC45 mRNA were determined by QRT-PCR and all measurements are shown relative to the expression levels of the β-actin housekeeping gene. Shown are the mean normalized values of three independent experiments, each in duplicates, using different cDNA samples.

### Extraction of RNA from formalin-fixed, paraffin-embedded tissues

Total RNA was extracted from the formalin-fixed, paraffin-embedded specimens as described [13]. Briefly, five to ten 6-μm sections were deparaffinized twice by addition of 1 ml of xylene and incubating at 55°C for 10 min, and dehydrated by subsequent washes with 100, 90 and 70% ethanol in RNase-free water. The pellet was then air-dried at room temperature, resuspended in 200 μl of RNA lysis buffer containing 20 mM Tris/HCL (pH 7.6), 20 mM ethylenediaminetetraacetic acid (pH 8.0), 2% sodium dodecyl sulfate (pH 7.2), and 500 μg/ml proteinase K, and incubated at 55°C for 16 hrs to dissolve the tissue. RNA was purified from the digested tissue using TRIzol reagent (Invitrogen, Carlsbad, CA, USA) according to the manufacturer's instructions. The RNA pellet was washed with 70% ethanol, air-dried, resuspended in 30–50 μl of RNase-free water, incubated at 55°C for 10 min, and stored at -80°C until use.

### Reverse transcription

RNA was reverse transcribed using First Strand cDNA Synthesis Kit (MBI Fermentas, Vilnius, lithuania) according to the manufacturer's introductions. In brief, 100 ng of total RNA, 1 μl of oligo(dT)18 primer (0.5 μg/μl) and DEPC-treated water in a final volume of 20 μl were mixed, incubated at 70°C for 5 minutes, and then chilled on ice. Four microliters of 5× Reaction buffer, 1 μl of ribonuclease inhibitor (20 IU) and 2 μl of 10-mM dNTP were added, and the tube was incubated at 37°C for 5 min. An additional 200 units of reverse transcriptase was added and incubated at 37°C for 60 min. The reaction was stopped by heating at 70°C for 10 min. The cDNA samples were stored at -20°C until use.

### Quantitative real-time PCR

Quantitative PCR was performed with the real-time PCR system, iQ™ 5 (Bio-Rad, Hercules, CA, USA), using SYBR Green I double strand DNA binding dye. The intron-spanning primers used for real-time PCR were designed to meet the specificity criteria using Oligo 6.0 software. The sequences of the PCR primer pairs and amplicon size of the selected genes are shown in Table 2.

**Table 2:** Primer sequences and amplicon size of the genes analyzed by QRT-PCR

**Gene name**	**Accession No**.	**Primer sequence**	**Amplicon size**
*β-ACTIN*	NM_001101.2	F: 5'-ATCCAGGCTGTGCTATCC-3'	105 bp
		R: 5'-GGCATACCCCTCGTAGAT-3'	
*CDC6*	U77949.1	F: 5'-TGTCAAAAGCCAGACTAT-3'	88 bp
		R: 5'-GTGAATAAGACCAACCCT-3'	
*CDC45*	AF053074.1	F: 5'-TGGACTGCACACGGATCT-3'	106 bp
		R: 5'-AACCTGGCTGCGGTATAG-3'	
*MCM2*	NM_004526.2	F: 5'-AGGCAGCATCCCCATTAC-3'	89 bp
		R: 5'-TCACATAGTCCCGCAGAT-3'	
*CDT1*	NM_030928.2	F: 5'-ATCCGCACCGACACCTAC-3'	135 bp
		R: 5'-TCTGAAGCCCACGTCTGT-3'	

The PCR mixture was prepared using the SYBR Green I Premix Ex Taq kit (TaKaRa Biomedical, Kusatsu, Japan) in a total volume of 20 ul containing 0.2 μmol of each primer, 10 μl of 2× SYBR Green I Premix Ex Taq, and 2 μl of cDNA. The reactions were performed in 8-well PCR strip tubes with flat caps. The initial template denaturation was at 95°C for 10 sec, followed by 45 two-step cycles of 5 sec at 95°C and then annealing and extension at 60°C for 20 sec. Fluorescence data were collected after each extension step. Melting curve analysis was performed to confirm the amplification specificity by heating the template over a 35°C temperature gradient at 0.5°C/sec from 60 to 95°C while continuously monitoring the fluorescence.

To demonstrate the linearity and efficiency and for the purpose of quantification, standard curves were generated. The housekeeping gene β-actin was used as the internal control. Gene specific PCR products were purified using a DNA extraction kit (MBI Ferments), quantified spectrophotometrically, and cloned into pMD18-T vector (TaKaRa). The sequences of the target genes were verified by DNA sequencing. Standard curves for β-actin, CDC6, CDT1, MCM2 and CDC45 were generated by performing serial dilutions (10^2^–10^6 ^copies) of the plasmids containing the target genes and amplifying them in every PCR run with the patient samples.

The relative expression level of the gene of interest in the patient samples was computed with respect to the internal standard. The normalized expression level of the target gene as obtained as follows: target copy number/β-actin copy number. Each sample was tested in duplicate, and each experiment was repeated at least twice using cDNA samples from separate reverse transcriptions. The generated data were averaged and expressed in relative units of normalized expression.

### Statistical analysis

One-Way ANOVA test and Kruskal-Wallis H test were used to determine differences in normalized mRNA expression levels between the precancerous and different squamous cell carcinoma clinicopathological parameters. The Mann-Whitney U test was used to assess pair-wise differences between the two cohort groups. A simple regression analysis was used to determine the relationship between the mRNA expression of different genes and the established clinicopathological parameters. Receiver operating characteristic (ROC) curves and the areas under the curves (AUC) were calculated to assess the accuracy of the tests. Differences between the areas under the curves were tested for statistical significance based on the estimated areas and their standard errors. A *p *value < 0.05 was considered statistically significant. Statistical analysis of results was carried out using SPSS v11.0 software.

## Results

**Figure 1:**
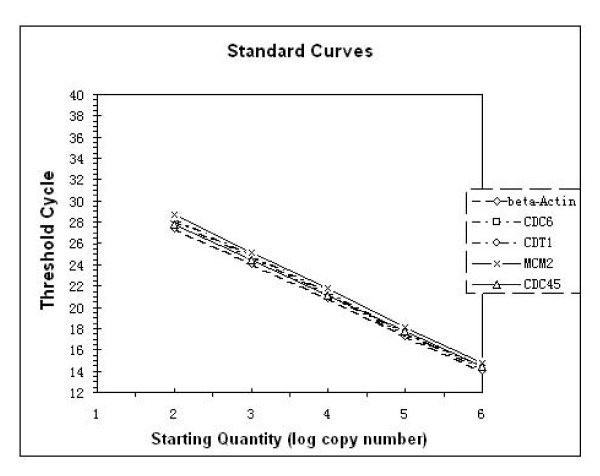
**CDC6, CDT1, MCM2, CDC45 and β-actin standard curves**. SYBR GREEN I standard curves for CDC6, CDT1, MCM2, CDC45 and β-actin were obtained by amplifying five serial dilutions of standards known concentration (10^2^–10^6^). The linearity of the plots shows the coefficient of correlation for five genes. R^2 ^> 0.994.

The mRNA levels of CDC6, CDT1, MCM2 and CDC45 in 31 cases of precancerous epithelial dysplasia and 33 cases of squamous cell carcinoma of the tongue from formalin-fixed, paraffin-embedded specimens were measured using QRT-PCR. The level of the target mRNA was normalized to the amount of β-actin mRNA in the same sample. Standard curves for β-actin and the target genes were shown in Figure 1. The linearity of the plots shows that the coefficient of correlation for all five genes was very high (R^2 ^> 0.994). The resultant target mRNA/β-actin ratio showed the normalized expression of the target gene. The normalized expression values and pathological variables are shown in Table 1.

### CDC6 quantitative real-time PCR analysis

**Figure 2:**
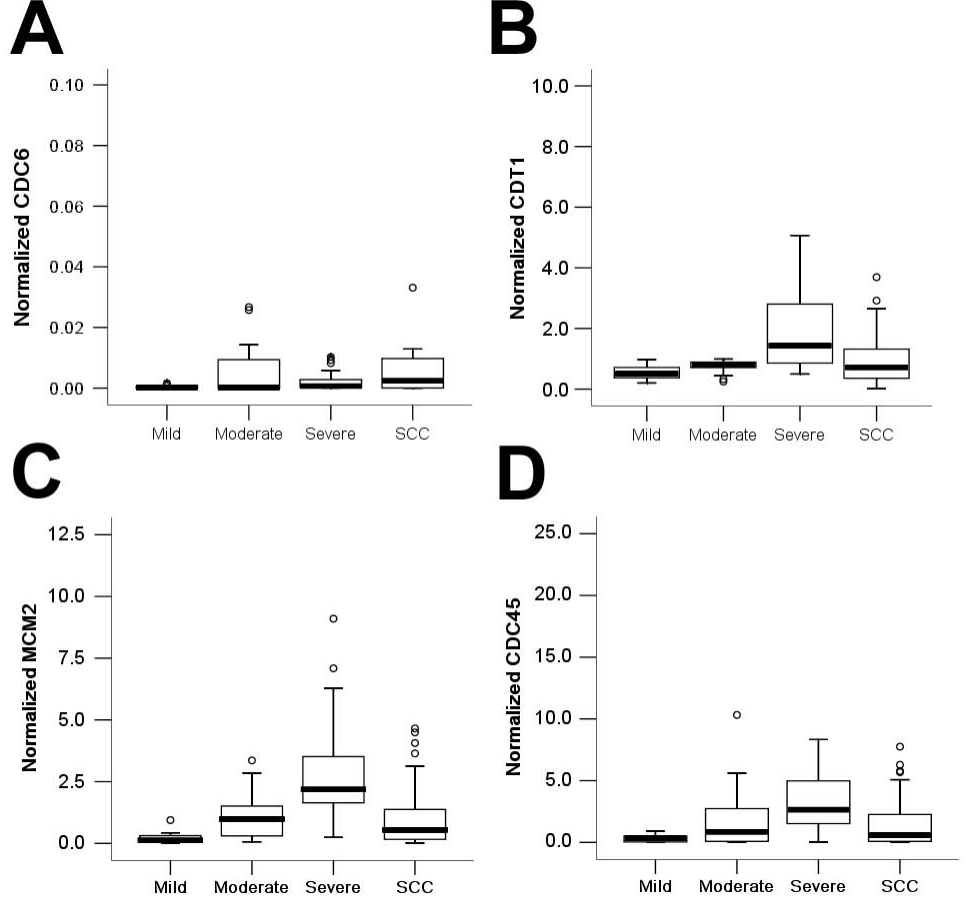
**Box plots for the normalized expression levels of CDC6 (A), CDT1 (B), MCM2 (C) and CDC45 (D) in mild, moderate and severe epithelial dysplasia and tongue SCC**. The box represents 50% quartiles (above 25% and below 75%) and the *solid line *within each box is the median gene expression value. The small circles represent the outliers.

Real-time PCR for CDC6 revealed that there was statistically significant overall difference in CDC6 expression among the mild, moderate and severe epithelial dysplasia and SCC of the tongue (*p *= 0.043; Kruskal-Wallis test) (Table 1; Figure 2A). In addition, the mean level of CDC6 was markedly higher in SCC of the tongue and severe epithelial dysplasia than in the mild epithelial dysplasia (*p *= 0.02 and 0.008, respectively; Mann-Whitney test). However, a statistically significant difference in CDC6 expression was not observed between the mild and moderate epithelial dysplasia (*p *= 0.912), between moderate and severe epithelial dysplasia (P = 0.451), between moderate epithelial dysplasia and SCC (*p *= 0.311), or between severe epithelial dysplasia and SCC (*p *= 0.728). Although the CDC6 mRNA increased with increasing grades of the clinicopathological classifications (except for the T-stage) of SCC, the Kruskal-Wallis test did not reveal statistically significant overall correlation of CDC6 mRNA level with tumor size (P = 0.159), lymph node status (*p *= 0.145), clinical stage (*p *= 0.242) or histological grade (*p *= 0.052). However, when using the Mann-Whitney test to assess pair-wise differences within each SCC classification group, statistically significant difference was found for N0 and N1 (*p *= 0.042), and for N0 and N2 (*p *= 0.031). These results showed that CDC6 mRNA was up-regulated during the malignant process of the tongue SCC.

### CDT1 quantitative real-time PCR analysis

There was statistically significant overall difference in CDT1 expression among the different epithelial dysplasia groups and SCC of the tongue (*p *= 0.034; Kruskal-Wallis test) (Table 1; Figure 2B). CDT1 mRNA expression levels were significantly higher in severe epithelial dysplasia than in mild epithelial dysplasia (*p *= 0.006), moderate epithelial dysplasia (*p *= 0.034), and SCC (*p *= 0.019). However, the differences between mild epithelial dysplasia and SCC (*p *= 0.202) and between moderate epithelial dysplasia and SCC (*p *= 0.72) did not reach statistically significant levels. Like CDC6, the ANOVA test revealed that there was no statistically significant overall correlation of CDT1 mRNA level with tumor size (*p *= 0.43), lymph node status (*p *= 0.200), clinical stage (*p *= 0.221) or histological grade (*p *= 0.215). With the advancement in the clinical stage and lymph node status of the SCC, CDT1 mRNA increased, but not to a statistically significant level of difference.

### MCM2 quantitative real-time PCR analysis

The overall difference in MCM2 mRNA expression among the different grades of the precancerous epithelia dysplasia and SCC of the tongue is highly statistically significant (*p *= 0.000; Kruskal-Wallis test) (Table 1; Figure 2C). MCM2 mRNA levels were significantly higher in SCC than in mild epithelia dysplasia (*p *= 0.000). In addition, the MCM2 expression level increased with increasing grade of dysplasia, with a highly significant difference between the mild and moderate epithelial dysplasia (*p *< 0.001), between the mild and severe epithelial dysplasia (*p *= 0.000), and between the moderate and severe epithelial dysplasia (*p *< 0.001). However, a statistically significant difference in MCM2 mRNA level was not observed between the moderate epithelial dysplasia and SCC (*p *= 0.35). The Kruskal-Wallis test revealed that there were statistically significant overall correlation of MCM2 levels with lymph node status (*p *= 0.007) and clinical stage (*p *= 0.002), but not with histological grade (*p *= 0.84) or tumor size (*p *= 0.127). When using the Mann-Whitney U-test to assess pair-wise differences within each SCC classification group, a statistically significant difference was observed between N0 and N2 (*p *= 0.006), N1 and N2 (*p *= 0.012), I and IV (*p *< 0.0001), I and III (*p *= 0.019), but not between N0 and N1 (*p *= 0.455), or II and III (*p *= 0.612).

### CDC45 quantitative real-time PCR analysis

The relationship between CDC45 mRNA and pathological variables is shown in Figure 2D. CDC45 mRNA level was significant lower in mild epithelial dysplasia than in the moderate epithelial dysplasia, severe epithelial dysplasia, and SCC (*p *= 0.008, < 0.001, and 0.026, respectively; Mann-Whitney test). The ANOVA F test revealed that there was no statistically significant overall correlation of CDC45 mRNA expression with tumor size (*p *= 0.085) or histological grade (*p *= 0.887). The P value for the lymph node status and clinical stage was 0.348 and 0.524, respectively, by using the Kruskal-Wallis test to analyze the overall correlation of CDC45 with these clinical parameters. When using the Mann-Whitney U-test to assess pair-wise differences within each SCC classification group, there was a statistically significant difference between N1 and N2 (*p *= 0.018), and between N0 and N2 (*p *= 0.004).

### Relationship of the expression of replication-initiation proteins with established clinicopathological parameters

**Figure 3:**
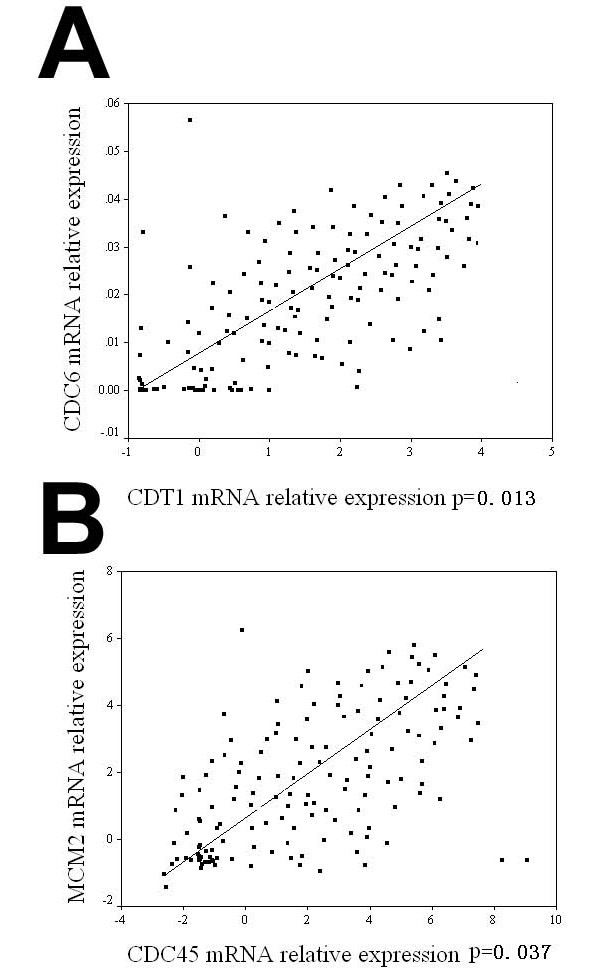
**Relationship between the expression of CDC6 and CDT1 (A), and of CDC45 and MCM2 (B) in dysplasia and tongue SCC**. The scatterplots suggested that the relationship could be modeled as linear. A significant correlation was observed (p = 0.013 and P = 0.037 respectively).

Table 3 depicts the relationship between the target gene expression and the established clinicopathological parameters. Significant correlations were observed for CDC6 (r = 0.323, *p *= 0.027), CDT1 (r = 0.538, *p *= 0.000), MCM2 (r = 0.592, *p *= 0.000) and CDC45 (r = 0.451, *p *= 0.001). We also used a simple linear regression analysis to assess the pair-wise relationship of different marker genes. Analysis revealed a highly significant linear relationship between CDC6 and CDT1 expression (r = 0.415, *p *= 0.013) (Figure 3A). In addition, a strong association was established between MCM2 and CDC45 expression (r = 0.244, *p *= 0.037) (Fig. 3B). But there were no correlation between other pair-wise genes (data not shown).

**Table 3:** Relationship between clinicopathological parameters and CDC6, CDT1, MCM2 and CDC45 mRNA expression

**Relationship**	**CDC6**	**CDT1**	**MCM2**	**CDC45**
correlation(*r*)	0.323	0.498	0.592	0.451
*p*-value	0.027	0.000	0.000	0.001

### Diagnostic accuracy of CDC6, CDT1, MCM2 and CDC45 mRNA expression levels

**Figure 4:**
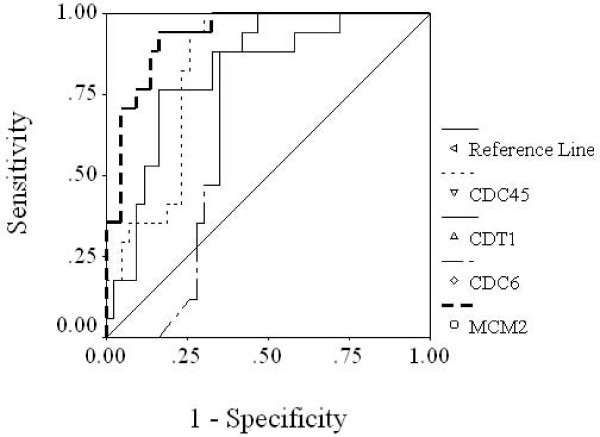
**Receiver operating characteristics (ROC) curve of normalized expression of CDC6, CDT1, MCM2, and CDC45**. Area under the curve (AUC) of 1.0 represents a "perfect" diagnostic test without any false negative or false positive results.

The receiver operating characteristic (ROC) curves of CDC6, CDT1, MCM2 and CDC45 were used to analyze the diagnostic accuracy based on the gene expression measured by QRT-PCR (Figure 4). The area under the ROC curve (AUC) was 0.679 for CDC6 (*p *= 0.032), 0.808 for CDT1 (*p *= 0.000), 0.933 for MCM2 (*p *= 0.000) and 0.836 for CDC45 (*p *= 0.000), indicating that MCM2 and CDC45 were superior to CDC6 and CDT1, and could be used as useful tumor markers in diagnosing tongue SCC. The accuracy of MCM2 with a sensitivity of 94.1% and specificity of 83.7% was highest, followed by CDC45 (sensitivity of 94.1% and specificity of 74.4%), CDT1 (sensitivity of 76.5% and specificity of 83.7%) and CDC6 (sensitivity of 88.2% and specificity of 63.1%). On the basis of Multi-ROC analysis, there was a statistically significant difference in pair-wise relationship between MCM2 and CDC6 (*p *< 0.01), MCM2 and CDT1 (*p *< 0.01), CDC6 and CDC45 (*p *< 0.05), and CDT1 and CDC45 (*p *< 0.05). However, a statistically significant difference was not observed between MCM2 and CDC45 (*p *> 0.05), or CDC6 and CDT1 (*p *> 0.05). In other words, MCM2 and CDC45 had a higher accuracy than CDC6 and CDT1 for distinguishing precancerous stages from malignant tongue SCC.

## Discussion

Alterations in cell cycle regulatory genes that lead to malignant transformation are one of the main hallmarks of neoplastic transformation. Logically the initiation of DNA replication represents a critical prerequisite for accurate cellular proliferation. Proteins that initiate DNA replication therefore represent excellent candidates as markers of the tongue squamous cell carcinoma. In this study, we found that CDC6, CDT1, MCM2 and CDC45 mRNA expression analyzed by quantitative real-time PCR are significantly higher in malignant SCC than mild precancerous epithelial dysplasia, and the expression levels in general increase with increasing grade of dysplasia.

In this study, the levels of CDC6 and CDT1 mRNA expression are up-regulated with increasing grade of dysplasia in SCC and correlate with clinicopathological parameters. Likewise, the mean level of CDC6 was markedly higher in SCC of the tongue than in the mild epithelial dysplasia. Our results showed that the tumor with higher CDC6 and CDT1 expression revealed more aggressive behavior. To our knowledge, CDC6 and CDT1 have not been previously studied in squamous cell carcinoma of the tongue. A simple linear regression analysis revealed a linear increase in the level of CDC6 mRNA expression in the mild, severe and SCC of the tongue, which demonstrated a linear relationship between increasing CDC6 protein expression and increasing severity of tongue dysplasia. Furthermore, our analysis revealed a strong correlation between CDT1 and CDC6 expression. It is known that CDT1 and CDC6 are downstream targets of E2F-1 to -3, which are capable of triggering the transcription of a reporter gene downstream of the CDC6 and CDT1 promoter [14,15]. Furthermore, transactivation of human CDC6, CDT1, Geminin and MCM7 is induced by the E2F-1 to -4 and not by the E2F-5 to -7 transcription factors [16]. Therefore, overexpression of CDC6 and CDT1 in SCC of the tongue may be due, at least in part, to an E2F-1 to -3 mediated effect.

Quantitative real-time PCR analysis showed that MCM2 mRNA expression is significantly higher in tongue SCC than in epithelia dysplasia. In addition, MCM2 expression is dependent of the grade of dysplasia, lymph node status and clinical stage. These observations are in agreement with previous studies in other tumors. For example, Kodani *et al *reported that overexpression of MCM2 was detected in dysplastic squamous epithelia cells, and the level increased with the advancement of dysplasia [17]. Similar results were obtained by Todorov *et al*. who examined a variety of human tumors and found that MCM2 is a useful diagnostic and prognostic marker [7].

In our study, a simple linear regression analysis revealed a highly significant linear relationship between the level of MCM2 mRNA expression in mild epithelia dysplasia, severe epithelia dysplasia and malignant tongue SCC. These findings clearly revealed that MCM2 overexpression correlated with the severity of dysplasia and associated with progression of lymph node and clinical stage in patients with tongue SCC. It may be a useful marker for evaluating tumor aggressiveness in tongue SCC.

Our results show that CDC45 mRNA is overexpressed in high grade dysplastic lesions and in tongue SCC. CDC45 overexpression has been reported in many different cancer types [18], but few studies have examined changes in CDC45 expression in the premalignant stage. We believe that this is the first study to show that CDC45 is frequently overexpressed in oral premalignancy.

Interestingly, a strong association was also established between MCM2 and CDC45 expression. It is similar to the relationship of CDC6 and CDT1. Both MCM2 and CDC45 are regulated by the upstream transcriptional factors E2F and cyclin D [19,20]. Studies in breast, lung, head and neck, esophagus, and colon mantle cell lymphoma found that E2F-1 overexpression is associated with increased proliferation and aneuploidy, and that cyclin D1 acts as a positive regulator of the transition from G_1_-phase to S phase by association with and activation of CDKs, leading to uncontrolled growth of tumor cells [21,22].

In this study, CDC6 expression was obviously lower than the other three genes. Similar difference in the protein levels of CDC6 and MCM2 was observed in cervical dysplasia [22]. These results may be, at least in part, resulted from the rapid turnover of CDC6 mRNA and protein which normally occurs in the cell.

The QRT-PCR assay described in this paper demonstrates high diagnostic sensitivity and accuracy. This assay is relatively rapid, technically amendable, and requires only a small amount of total RNA extracted from formalin-fixed and paraffin-embedded specimens. Furthermore, QRT-PCR test has the potential for automation and standardization. Therefore, the assay has potential clinical utility as a complementary technology to the current pathological diagnosis methods in distinguishing malignant SCC from benign dysplasia.

## Conclusion

This study shows that mRNA of the replication-initiation proteins are overexpressed in tongue dysplastic lesions and SCC and that the higher levels are associated with lesions that are more likely to progress to tongue SCC. These proteins are useful diagnostic markers for distinguishing malignant from benign epithelial dysplasia and for early detection and diagnosis of SCC as an adjunct to clinicopathological parameters.

## Abbreviations

SCC: squamous cell carcinomas; QRT-PCR: quantitative real-time polymerase chain reaction.

## Competing interests

The authors declare that they have no competing interests.

## Authors' contributions

JNL carried out and coordinated the study, conducted QRT-PCR and data analysis, and drafted the manuscript. CL, YJL and ZT participated in the background work, study design, interpretation of data, and revision of the manuscript. CJF, HJL, and GQL were involved in the sample acquisition, sample selection, and clinical data acquisition. All authors reviewed and commented on successive drafts of the manuscript and approved the final manuscript.

## Pre-publication history

The pre-publication history for this paper can be accessed here:


